# Edoxaban Overdose in a Child: Unexpected Observation of Clot Lysis

**DOI:** 10.1055/a-2769-7862

**Published:** 2025-12-30

**Authors:** Mouna Sassi, Emna Sfar, Linda Khefacha, Nouha Berrayana, Radhia Haj Salem, Slaheddine Chouchane

**Affiliations:** 1 Hematology Laboratory of Maternity and Neonatal Center of Monastir, Faculty of Pharmacy of Monastir, Monastir, Tunisia; 2 Pediatric Department, Fattouma Bourguiba University Hospital, Faculty of Medicine of Monastir, Monastir, Tunisia

## Background

Direct oral anticoagulants (DOACs) are increasingly prescribed in adults but remain exceptional in pediatrics. Reports of overdose in children are very rare, and clinical data on management are limited. 1 2 3 Edoxaban is a direct factor (F) Xa inhibitor, inhibiting free and bound FXa. 4 We present here the first description, to our knowledge, of edoxaban overdose in a child, in whom we observed an unusual clot-lysis phenomenon that may suggest a novel mechanism of action.

## Clinical Case Summary and Management

A 21-month-old girl (12 kg) presented to our tertiary pediatric department 4 hours post-accidental ingestion of probable 3 pills of edoxaban 60 mg (180 mg). Edoxaban was prescribed to her father to prevent the recurrence of thrombosis. On presentation, the clinical examination was normal. No hemorrhagic sign was identified. Initial blood tests showed normal full blood count and normal renal and liver function (creatinine 21 µmol/L, aspartate aminotransferase 28 IU/L, alanine aminotransferase 20 IU/L). However, the coagulation profile at H + 4 was abnormal: prothrombin time (PT) was prolonged at 25.4 seconds, with an international normalized ratio (INR) of 2.2, consistent with significant systemic absorption of edoxaban.

The absence of bleeding was reassuring but did not exclude toxicity. Edoxaban absorption is rapid (1–3 hours), so the maximum changes are generally observed 2 to 4 hours after its intake. 5 As gastric decontamination was deemed too late, management focused on enhancing clearance. Edoxaban half-life is 10 to 14 hours in patients with normal renal function. More than two-thirds of urinary elimination occurred within 8 hours. 6 Therefore, the child was started on intravenous hyperhydration at 3 L/m ^2^ /day. At H + 8, prothrombin complex concentrate (PCC, 25 IU/kg) was administered as a precautionary measure.

## Prothrombin Time

The child was followed up with PT, which normalized when assessed at H + 24 (PT 12.4s/ INR 1.1). This result likely reflected accelerated elimination in children with preserved renal function. Edoxaban had dose-dependent effects on PT, but reagents vary significantly in their sensitivities. 6 7 PT can provide qualitative information if the anti-Xa assays are not available.

## Anti-Xa Activity

Edoxaban monitoring with anti-Xa activity and viscoelastic tests was performed at H + 24, H + 48, H + 72, and H + 120. Edoxaban levels were estimated from an anti-Xa chromogenic assay using ACL TOP 550 analyzer (Instrumentation Laboratory, Bedford, MA), HaemosIL anti-Xa liquid reagent with a calibrator for unfractionated heparin (UFH) and low-molecular-weight heparin (LMWH). Anti-Xa activity was measured using a UFH/LMWH-calibrated chromogenic assay, acknowledging the known limited accuracy of this method for edoxaban at low concentrations due to the lack of dedicated calibrators. 2 8 9 Nevertheless, the detectable level at H + 24 and subsequent undetectable values confirmed rapid elimination, consistent with reported pediatric pharmacokinetics. 10 11

## Viscoelastic Tests (ROTEM)

ROTEM delta analyzer (Instrumentation Laboratory, Werfen, Barcelona, Spain) was used to perform viscoelastic tests with EXTEM, INTEM, FIBTEM, and APTEM reagents. Studied parameters were clotting time (CT), maximum clot firmness (MCF), and maximum lysis (ML). A prolonged CT signifies a clinically relevant edoxaban effect on FXa, 12 unlike the MCF, which remains constant. 6 However, CT-EXTEM is only affected if the plasma concentration of edoxaban is greater than approximately 100 ng/mL. 13

**Fig. 1:**
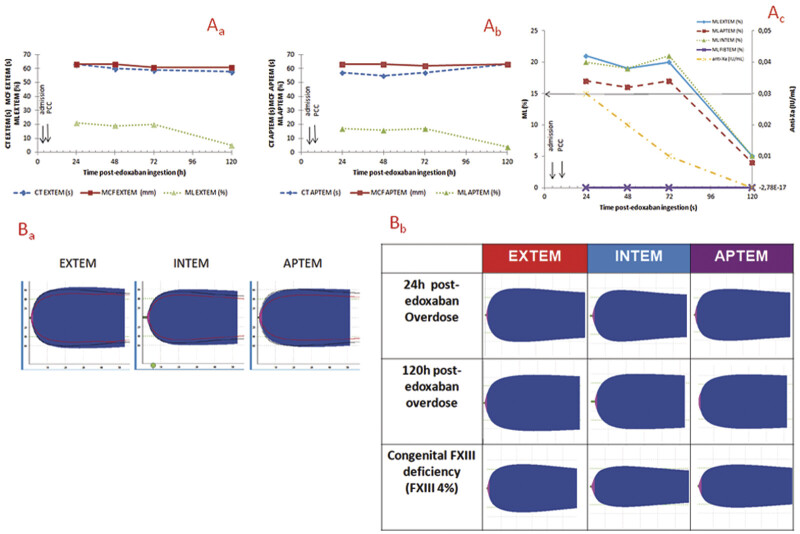
Edoxaban effects in ROTEM tests and parameters at different times after accidental ingestion of 180 mg edoxaban in a 21-month-old child. (A) Graphs of variation of ROTEM parameters over 120 hours post accidental ingestion of 180 mg edoxaban in a 21-month-old child. ( **a** ) Edoxaban effects on EXTEM parameters (clotting time [CT]; maximum clot firmness [MCF], maximum lysis [ML]); ( **b** ) endoxaban effects on APTEM parameters (CT, MCF, ML); ( **c** ) endoxaban effects on ML and anti-Xa activity. ( **B** ) ROTEM profiles post accidental ingestion of 180 mg edoxaban in a 21-month-old child. ( **a** ) Superposition of Rotem profiles: red profiles represent congenital FXIII deficiency (FXIII 4%); black profiles represent 24-hour post-edoxaban overdose; colored profiles represent 120-hour post-edoxaban overdose; ( **b** ) comparison of ROTEM profiles between two different times after edoxaban ingestion (H + 24 and H + 120) and congenital FXIII deficiency (same patient but different sample from that of B _a_ ).

From the first ROTEM performed at H + 24, no anomalies were observed either on the CT or on the MCF, which is not surprising given the value of anti-Xa activity ( Fig. 1Aa,b ). CT is not sensitive enough to quantify DOACs compared to the recommended anti-Xa activity, which can quantify low edoxaban plasma concentrations better than CT. So, CT results within the normal range do not exclude residual DOACs plasma levels. 12 Interestingly, ROTEM revealed unexpected findings. Although CT and MCF remained within acceptable limits, there was a persistent increase in ML, up to 21% across EXTEM, INTEM, and APTEM assays during the first 3 days ( Fig. 1Ac ). This exceeded the usual threshold for hyperfibrinolysis (ML >15%) but decreased by H + 120. However, this increase in ML was not recorded with FIBTEM. We hypothesized that the observed reduction in clot strength and firmness registered with EXTEM, INTEM, and APTEM tests may result from enhanced clot retraction or destruction. Clot-lysis effect was not related to fibrinolysis. Indeed, the addition of aprotinin (APTEM) did not alter results, excluding hyperfibrinolysis as the cause. Similar to adults, in children, fibrinogen concentrations, platelet count, and FXIII contribute to clot firmness. 14 Although ML above 15% was highlighted in some children under 2 years old without increased bleeding tendency, 15 what is original in our case is the important decrease in ML coinciding with a total disappearance of any anti-Xa activity.

Surprisingly, the child's ROTEM profiles until H + 72 mimic, in a certain way, that of FXIII deficiency when we compared our case (FXIII 81%) with an age- and sex-matched FXIII-deficient child (FXIII 4%; local laboratory profiles not yet published) ( Fig. 1Ba,b ). This striking finding seems likely to be due to FXIIIa deficiency. FXIIIa stabilizes the fibrin clot in the presence of calcium. FXIII is activated by thrombin but edoxaban suppresses thrombin generation through the propagation phase triggered by the prothrombinase complex.

This interpretation is consistent with experimental data showing that FXa inhibition can suppress FXIII activation and produce thinner, less stable fibrin networks with enhanced susceptibility to fibrinolysis. 16 17 Conversely, clinical fractal-analysis data in adults treated with apixaban demonstrated prolonged clotting kinetics without significant change in fibrin microstructure, indicating that FXa inhibitor effects on clot stability may vary by dose, biological context, and assay modality. 18 In addition, recent in-vitro work demonstrates that FXa contributes to endogenous profibrinolytic activity and that high concentrations of apixaban can impair plasmin generation and delay clot formation, reinforcing a concentration-dependent effect of FXa inhibition on fibrinolysis dynamics. 19

Collectively, these mechanistic data support the interpretation that transient, low-level edoxaban exposure may alter clot architecture through reduced FXIII activation and subtle modifications of fibrin network integrity.

## Outcome

The child remained clinically stable without bleeding and was discharged at H + 120. This evolution is consistent with previously reported benign outcomes in pediatric DOAC exposures and therefore confirms, rather than extends, existing safety knowledge.

## Conclusion

This case confirms that accidental edoxaban overdose in a healthy child can remain clinically silent with rapid normalization of coagulation tests. Beyond the benign clinical course, the key finding is the reproducible pattern of increased clot lysis on viscoelastic testing, mimicking FXIII functional impairment despite negligible anti-Xa activity. This observation suggests a potential indirect effect of edoxaban on clot stabilization at low plasma levels. Further studies are needed to clarify this mechanism and determine its relevance in pediatric anticoagulation monitoring.
